# How to Fight an Infodemic: The Four Pillars of Infodemic Management

**DOI:** 10.2196/21820

**Published:** 2020-06-29

**Authors:** Gunther Eysenbach

**Affiliations:** 1 JMIR Publications Toronto, ON Canada

**Keywords:** infodemiology, infodemic, COVID-19, infoveillance, pandemic, epidemics, emergency management, public health

## Abstract

In this issue of the *Journal of Medical Internet Research*, the World Health Organization (WHO) is presenting a framework for managing the coronavirus disease (COVID-19) infodemic. Infodemiology is now acknowledged by public health organizations and the WHO as an important emerging scientific field and critical area of practice during a pandemic. From the perspective of being the first “infodemiolgist” who originally coined the term almost two decades ago, I am positing four pillars of infodemic management: (1) information monitoring (infoveillance); (2) building eHealth Literacy and science literacy capacity; (3) encouraging knowledge refinement and quality improvement processes such as fact checking and peer-review; and (4) accurate and timely knowledge translation, minimizing distorting factors such as political or commercial influences. In the current COVID-19 pandemic, the United Nations has advocated that facts and science should be promoted and that these constitute the antidote to the current infodemic. This is in stark contrast to the realities of infodemic mismanagement and misguided upstream filtering, where social media platforms such as Twitter have advertising policies that sideline science organizations and science publishers, treating peer-reviewed science as “inappropriate content.”

## The World Health Organization Declares an Infodemic and Crowdsources a Framework

In this issue of the *Journal of Medical Internet Research*, a high-profile group of authors associated with the World Health Organization (WHO) have published a paper entitled “Framework for Managing the COVID-19 Infodemic: Methods and Results of an Online, Crowdsourced WHO Technical Consultation” [1]. In the paper, the authors collected and organized global ideas to fight the current coronavirus disease (COVID-19) infodemic declared by the WHO on February 15, 2020. Impressively, this consultation meeting was entirely conducted online, and, as noted by the authors, turned out to be one of the largest meetings ever convened by the WHO.

In my capacity as the Editor of the *Journal of Medical Internet Research*, I had the honor of attending the meeting as an invited panelist to talk about the responsibility and potential approaches for scholarly publishers to combat the infodemic. A summary of my presentation and a project proposal for scientists, editors, and science communicators will be the subject of a separate, forthcoming editorial.

I also attended the meeting as one of the early researchers in this field. In fact, I coined the terms *infodemiology* [2,3], *infodemic*, and *infoveillance* [4] over the course of the past 20 years. It is not without pride that I witnessed how this line of research is now formally acknowledged by public health organizations and the WHO as a novel, emerging scientific field and critical area of practice during a pandemic [1]. It should also be noted that this journal has been a pioneer in encouraging and disseminating this line of research and has provided a central forum for researchers to discuss and publish their infodemiology work in a high-impact journal [5-7].

## A Brief History of Infodemiology

Although the term *infodemiology* was coined in 2002 [2], concerns over infodemics or outbreaks of misinformation are almost as old as the World Wide Web itself. A widely cited paper published in 1997 in the *BMJ* drew attention to the, now seemingly trivial, finding that medical information found on the internet is not always reliable [8]. This paper sparked an avalanche of publications describing and analyzing the quality of medical information on different topics, which I, together with my colleagues, synthesized in a comprehensive systematic review published in *JAMA* in 2002 [9]. At the time, I was also leading major EU-funded projects (MedCERTAIN and MedCIRCLE) attempting to label health information on the internet with machine-readable metadata [10,11], an ambitious project with the goal to create something akin to a digital “immune system” for misinformation [12], with distributed descriptive and evaluative metadata as machine-processable “antibodies.” The metadata (evaluative or descriptive, supplied by third parties or the providers themselves) are perhaps conceptually comparable to contemporary efforts by Facebook and Twitter to fact-check information and label social media posts that are problematic [13], although these tags are not necessarily machine processable and cannot be harvested by third-party applications, which was the underlying idea of the MedCERTAIN/MedCIRCLE projects [10,11]. The goal was to create a global infrastructure for such machine-processable annotations that would allow humans and machines to attain a more complete picture about what other people and institutions have to say or think about a certain information provider or piece of information. The vast amount of information on the web, the dynamic nature of the web, and questions on the scalability of this approach were obvious limitations, but, perhaps it is time, for the sake of future infodemic preparedness, to revisit some of these “semantic web” ideas articulated over 20 years ago and to combine them with today’s powerful artificial intelligence tools, because, given the advances in natural language processing, many of these metadata labels could now be generated automatically. My former project partner Dan Brickley is now working with Google and is running schema.org [14], which organizes community vocabularies to enable such applications.

I coined the term “infodemiology” in 2002 in a short guest editorial in the *American Journal of Medicine* [2], defining “infodemiology” as a “new emerging research discipline and methodology” comprising the “the study of the determinants and distribution of health information and misinformation—which may be useful in guiding health professionals and patients to quality health information on the Internet.” Equipped with the awareness that “quality of health information” as well as “misinformation” is often hard to define (as quality is in the eye of the beholder, and a “fact” in medicine requires more than one patient or study), I framed infodemiology as a method to “identify areas where there is a knowledge translation gap between best evidence (what some experts know) and practice (what most people do or believe)” [2]. While this early work focused on information *supply* (what is published on the internet), I added, in 2006, analysis of information *demand* (search queries) to the concept, realizing that harvesting what people are searching for on the internet could inform areas of public health such as surveillance. I illustrated this with demonstrating the predictive power of Internet searches to predict flu outbreaks [3,4]; an idea that inspired Google Flu Trends [15]. With the emergence of Twitter, more “social listening” infoveillance studies became possible, and H1N1 (SwineFlue) became the first pandemic where this approach could be demonstrated; my graduate student Cynthia Chew and I, analyzed the content of pandemic tweets and determined, among other interesting findings, a prevalence of misinformation of 4.5% [16].

In a tweet posted on April 14, 2020, Secretary General of the United Nations (UN) António Guterres announced a UN communications response initiative countering the infodemic with facts and evidence [17]; however, what we have learned in 20 years of infodemiology research is that the quality of health information is an elusive concept, as in medicine, the truth is not always easy to determine, especially in a rapidly evolving situation.

While certain technical quality criteria, readibility scores, and the compliance with ethical quality criteria (such as the presence of disclosure of who owns the site and conflicts of interests, all aspects that are important to determine the source credibility) are relatively easy to measure, the concepts of *accuracy, facts*, and *truth* usually require the presence of evidence-based guidelines or systematic reviews as a gold standard to determine what works and what does not. In a rapidly evolving situation such as the COVID-19 pandemic, some of the problems are the rapid rate of new scientific information published and the inability of researchers, policy makers, journalists, and ordinary citizens to keep up with quickly changing facts. In other words, the current pandemic is partly a challenge to filter (in real time) the sheer quantity of information published on a daily basis. The founder of the website Retraction Watch, Ivan Oransky, stresses that science is a conversation [18]. Even a publication of a clinical study is not the last word and studies may be contradicted or proven wrong. In the early phases of a pandemic, "facts" are perhaps more accurately referred to as "BETs" (best evidence at the time). Facts are sparse and recommendations based on BETs are subject to change. The COVID-19 pandemic has illustrated this with examples such as mask-wearing recommendations, use of certain drugs such as hydroxychloroquine, and social distancing or school opening guidelines. The public health and medical evidence also needs to be integrated with economic and political considerations and may be subject to cultural variations and influences. Thus, the proposal to fight the infodemic by spreading “facts” is easier said than done when it is not clear what the exact facts are.

## The Information “Cake” Model

The following model is not the framework presented in the WHO paper in this journal. It is an "expert opinion" (to the degree as somebody - and probably the first - who has "Infodemiologist" in his LinkedIn profile can be considered an "expert"). It supplements the WHO framework by providing a first broad roadmap on how to fight an infodemic. 

The current infodemic is a crisis to distill the sheer quantity of information, which is occurring on four levels: (1) science, (2) policy and practice, (3) news media, and (4) social media.

**Figure 1:**
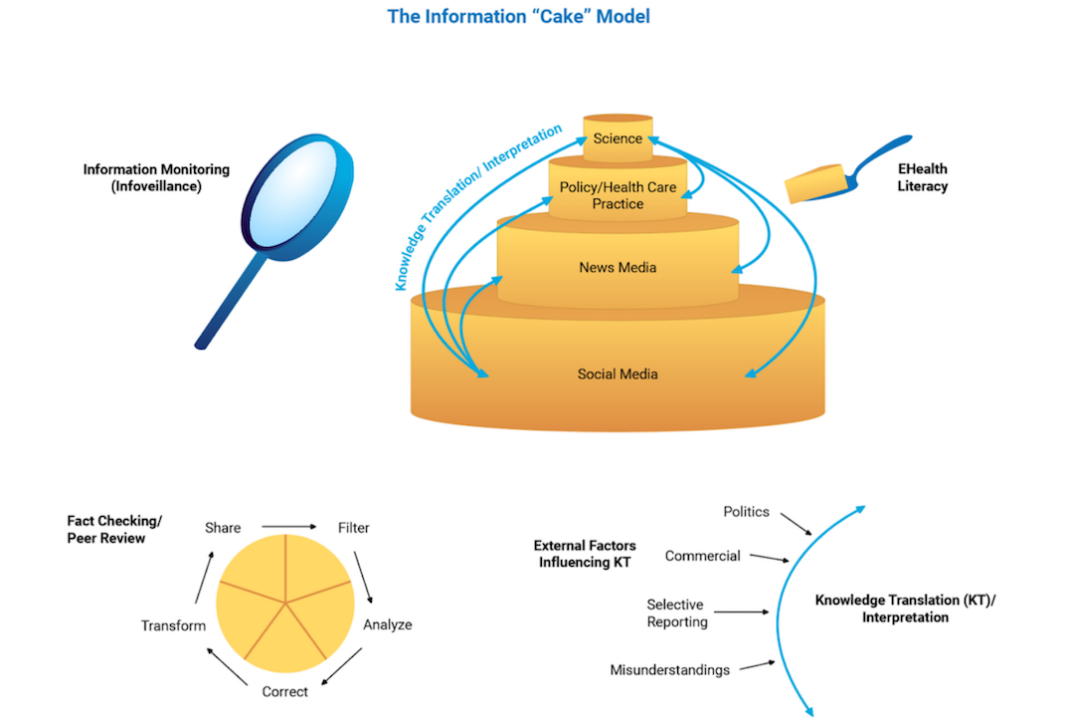
The Information “Cake” Model. The four pillars of infodemic management are information monitoring (infoveillance; top left); building eHealth Literacy and science literacy (top right); encouraging knowledge refinement and quality improvement processes for information providers, such as fact checking and peer review (bottom left); and Knowledge Translation, meaning to translate knowledge from one layer to another, while minimizing distorting factors (bottom right). eHealth: electronic health; KT: knowledge translation.

The wedding cake model (Figure 1) illustrates these four levels as layers. The size of the layers is proportional to the amount of information generated by these four groups of actors. The model also shows some information flows and knowledge translation activities that take place between these different levels. Science is the smallest layer of the wedding cake in terms of the amount of information, and it is depicted at the top of the information wedding cake, which represents rigorous and selective information production cycles. Clearly, misinformation can be found here as well, perhaps measured by the number of retractions, which, as of June 2020, stands at less than two dozen retracted articles [19], but this number is certain to increase. With over 26,000 COVID-19 articles indexed in Pubmed, this represents less than 0.1% of the published research, even though there may be a higher rate within the segment of unreviewed preprints, some of which may never see the light of journal publication, which may be another metric for the prevalence of scientific misinformation (somebody please do a study on this and submit it to the *Journal of Medical Internet Research*!). The main problem is not so much the prevalence of misinformation in the science layer, but the challenge of translating this information into actionable recommendations and conveying conclusions for different audiences and stakeholders in other layers, illustrated by the knowledge translation arrows in Figure 1.

Social media is depicted as the largest and last segment of the wedding cake, representing the vast amount of nearly unfiltered and uncontrolled information generated or amplified by the public. Information in social media is, of course, also generated by science organizations, policy makers, health care organizations, and journalists.

## First Pillar: Facilitate Accurate Knowledge Translation

Between the levels, knowledge translation processes need to take place to translate the information from one audience to another, and these knowledge translation processes are, perhaps, the main mechanisms where information becomes misinformation, as the interpretation of “facts” is subject to multiple potentially influencing factors such as politics, commercial interests, selective reporting, and misunderstandings. These knowledge translation processes take place between all four layers of the cake, for example, between public health policy recommendations and news media (to keep the figure simple, not all possible knowledge translation arrows between the different layers are shown in Figure 1). *The first pillar of infodemic management is to support, facilitate, and strengthen accurate knowledge translation*. In the WHO paper hint at that when they write “knowledge should be translated into actionable behaviour-change messages presented in ways that are understood by and accessible to all individuals” [1], but this is of course just one example for a KT problem, while there are also other KT challenges, e.g. between science and policy makers. Realizing that knowledge translation is subject to political, commercial, or other influences that distort the scientific message, the influencing factors should be minimized or, if present, at least clearly disclosed and called out.

## Second Pillar: Knowledge Refinement, Filtering, and Fact-Checking

The FACTS model [20] depicted as an insert in Figure 1 reminds us that within each layer, there are knowledge refinement processes such as fact checking and quality assurance mechanisms at play, which are sometimes visible and sometimes invisible to the end user. For example, on the science level, the process of peer reviewing and publishing scholarly work is a method to constantly filter, refine, and improve the information generated by previous scholars. This also means that within each layer, there is a continuum ranging from raw, tentative, and possibly problematic information to highly refined and trustworthy information. *The second pillar of infodemic management is to encourage, facilitate, and strengthen knowledge refinement and filtering processes on each level, to accelerate internal quality improvement processes.* Within each layer, information in different stages of this "refinement" process can be found (for example, in the science layer, unreviewed preprints, laying right next to peer-reviewed scholarly communications); thus, clearly labelling the provenance of the information at the different knowledge production stages is equally as important as facilitating and accelerating them..

## Third pillar: Build eHealth Literacy

The cake-serving utensil illustrates that in the age of the internet and openness, the end user is able to (but not always equipped) to consume information from any level, in any refinement stage, making eHealth literacy an essential skill in a networked world. eHealth literacy is defined as "the ability to seek, find, understand, and appraise health information from electronic sources and apply the knowledge gained to addressing or solving a health problem." [21,22]. In the information age (which is now perhaps shifting to the infodemic age!), the user carries a significant part of the responsibility to select and downstream-filter trustworthy health information. For example, nothing stops a user from tapping into the vast array of unreviewed preprints published in preprint servers such as medRxiv, but interpreting and contextualizing the information found here requires significant eHealth literacy (which includes science literacy) skills. Thus, *the third pillar of infodemic management is to enhance the capacity of all stakeholders to build eHealth literacy, to select and assess health and science information found on the different layers of the information cake.* This aspect is notably underdeveloped in the WHO paper’s taxonomy but can be seen as part of WHO’s “identify evidence” category.

## Fourth Pillar: Monitoring, Infodemiology, Infoveillance, and Social Listening

The fourth pillar of infodemic management is continuous monitoring and analysis of data and information exchange patterns on the internet, a field I have called *infodemiology and infoveillance* [4]. My idea was that similar to surveillance in pandemics we want to be able to detect outbreaks of misinformation, rumors, falsehoods, to counter them with facts or other interventions. Infoveillance requires generating metrics on information supply on the internet, including its quality (for example incidence of anti-vaccination tweets), as well as information demand metrics, such as search queries or questions posed on social media or other web 2.0 platforms. In Figure 1, infoveillance is illustrated as a lens magnifying the information exchange patterns within different communities and for different subtopics.

## Conclusions

Poorly executed and uncoordinated infodemic management may lead to unintended consequences such as the sidelining and suppression of science in favor of political and commercial interests.

Such an unintended consequence is demonstrated, for example, by the poorly thought-through advertising policy of Twitter [23], which only allows governments and selected news media, but not science organization or science publishers, to amplify messages. Under this “inappropriate content” policy of Twitter, only the following kinds of tweets are allowed to be amplified and promoted [23]:

- Public service announcements related to COVID-19 from governments and supranational entities (for example, World Health Organization) as well as trusted partners approved by the Public Policy team  
- News related to COVID-19 from media publishers who have been exempted under the Political Ads policy.

Notably missing from Twitters' exemption list are science organizations and science publishers. Is this indicative of a sidelining of science in favor of politics, or just an oversight? JMIR Publications (as science publisher) ran into this problem first-hand when we were prevented by Twitter from disseminating COVID-19 peer-reviewed research, promoting our virtual COVID-19 preprint journal clubs, etc. Therefore, if the UN declares an infodemic and promotes science and “facts” as the antidote, then the suppression of science as “inappropriate content” by private social media platforms should be an alarming sign that indicates that there is ample room of improvement in how the current infodemic is managed and coordinated by different stakeholders.

The current COVID-19 pandemic is a 9/11 for public health, but also an opportunity to develop and formalize tools and approaches for future infodemic management. It is also an opportunity to re-engineer certain knowledge refinement processes such as scholarly publishing and peer-review (stayed tuned to what we are doing with JMIRx.org). Much as improvements in information flows between government agencies post-9/11 helped to prevent another major act of terrorism in the United States, improved and preventive infodemic prevention and management can mitigate the next infodemic, which we will face as soon as a vaccine is available. The price for freedom of speech and improved information technology is an increased susceptibility to infodemics. We are entering the age of infodemics. 

## Abbreviations

BET: Best evidence at the time
COVID-19: coronavirus disease
eHealth: electronic health
UN: United Nations
WHO: World Health Organization
